# Comparative efficacy and safety of first-line treatments in patients with metastatic renal cell cancer: a network meta-analysis based on phase 3 RCTs

**DOI:** 10.18632/oncotarget.7511

**Published:** 2016-02-19

**Authors:** Xiaofeng Chang, Fan Zhang, Tieshi Liu, Rong Yang, Changwei Ji, Xiaozhi Zhao, Linfeng Xu, Guangxiang Liu, Hongqian Guo

**Affiliations:** ^1^ Department of Urology, The Affiliated Nanjing Drum Tower Hospital, Medical School of Nanjing University, Nanjing, China; ^2^ Jiangsu Key Laboratory of Molecular and Functional Imaging, Department of Radiology, Zhongda Hospital, Medical School, Southeast University, Nanjing, China; ^3^ Medical School, Southeast University, Nanjing, China; ^4^ Collaborative Innovation Center of Suzhou Nano-Science and Technology, Suzhou Key Laboratory of Biomaterials and Technologies, Suzhou, China

**Keywords:** metastatic renal cell carcinoma, efficacy, safety, therapy, meta-analysis

## Abstract

It is impossible to conduct head-to-head trials of all the therapies to determine optimal treatment in the rapidly advancing era of therapies for metastatic renal cell carcinoma (mRCC). In this network meta-analysis,we aimed to compare efficacy and safety of first-line treatments for mRCC. We searched PubMed, Embase, the Cochrane Central Register of Controlled Trials, and unpublished studies were also sought through “clinicaltrials.gov” from their inception through January 31, 2016. A database search identified 1253 articles, with 11 studies meeting the eligibility criteria. A total of 7597 patients in twelve different treatment arms were assessed. Network meta-analysis showed sunitinib had a significantly longer PFS than IFN-α (SMD=−5.68; 95%CI: −10.76,−0.86; *P*<0.001) and placebo (SMD=−6.71; 95%CI: −12.65,−0.79; *P*<0.001), meanwhile, pazopanib had a significantly longer PFS compared with placebo (SMD=5.13; 95%CI: 0.43, 10.09; *P*<0.001). The cumulative ranking probability curve indicated that sunitinib had the highest probability of being the best treatment modality in terms of PFS and it also had the highest probability of being the safest drugs as the first-line treatment when it came to SAE. Thus, sunitinib might be the best choice of first-line treatment for patients with mRCC because it has the most favorable balance between efficacy and safety.

## INTRODUCTION

Renal cell carcinoma (RCC) accounts for 85%90% of primary renal malignant tumors, which results in more than 120 000 new cases in Europe and the USA every year, and causes about 60 000 deaths[1]. Most of these cases are clear-cell renal cell carcinomas [2]. Up to 30% of patients have metastases at the time of the initial diagnosis, resulting in poor prognosis and subsequent 5-year survival rate of only 12% [3] Until 2005, only interleukin-2 (IL-2) was approved by FDA to be used in the systemic therapy of mRCC, and occasional and continuous complete response could be achieved when high-dose IL-2 was administrated. Interferon-alfa (IFN-α) is another cytokine therapy which is also widely used in the systemic therapy of mRCC. A meta-analysis based on randomized controlled trials (RCTs) has shown that patients with mRCC could benefit modest response rate from IFN-α [4].

The greater and deeper understanding of the mechanisms involved in the pathogenesis of mRCC led to development of more promising target treatment options [5, 6]. Previous RCTs of targeted therapies *versus* cytokine therapies or placebos manifested that targeted therapies showed superiority in PFS, response, and tolerability [7, 8]. Since 2005, several targeted drugs in treating mRCC have been approved by FDA. Even though most of patients with mRCC could benefit a lot from these targeted therapies, little continuous response has been reported. Numerous RCTs are ongoing to evaluate new drugs or therapeutic schedules for mRCC; however, it is impossible to conduct head-to-head evaluations of all the therapies to determine optimal treatment in the rapidly advancing era of targeted therapies. Given this, we reviewed all the RCTs and conducted a network meta-analysis to provide a clinically useful summary of the results that can be used to guide treatment decisions.

## RESULTS

### Study characteristics

**Figure 1:**
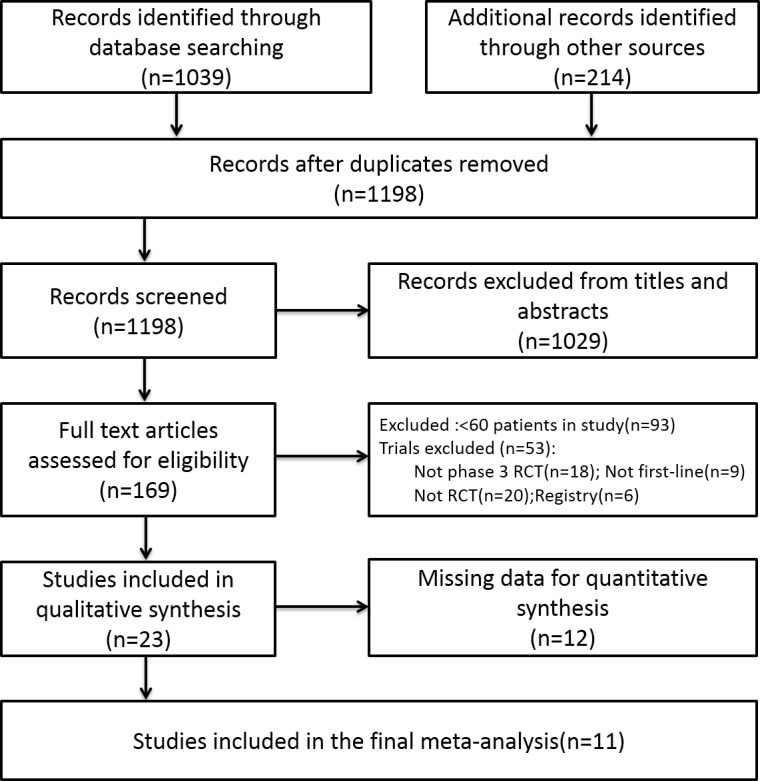
Flow chart of study selection

**Figure 2:**
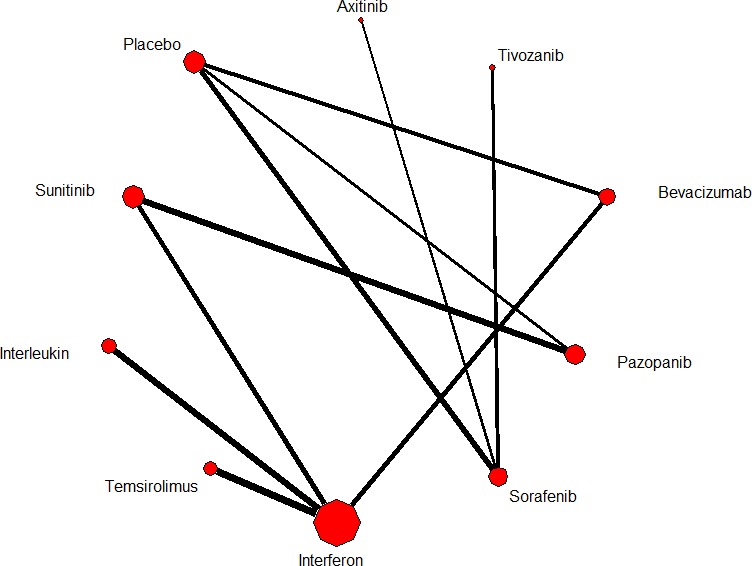
Network of eligible comparisons for the multiple-treatment meta-analysis for efficacy (progression free survival) Each link represents at least 1 study and the widths of each link are proportional to the number of studies comparing the particular arms. The size of each node is proportional to the total sample size.

Our search strategy yielded 1253 potential relevant studies, of which 169 potential eligible articles were analyzed and 158 reports that did not meet eligibility criteria were excluded from our analysis (Figure 1). Thus, there were altogether 11 studies up to January 2016 found to be eligible for the final network meta-analysis [9-19]. Table 1 summarizes the characteristics of the included trials. A total of 7597 patients in twelve different treatment arms were assessed: IFN-α alone, temsirolimus alone, sorafinib alone, sunitinib alone, pazopanib alone, axitinib alone, tivozanib alone, IFN-α+IL-2 + fluorouracil, bevacizumab + IFN-α, IFN-α+placebo, temsirolimus+bevacizumab and placebo. Figure 2 shows the network of eligible comparisons for the network meta-analysis.

**Table 1:** Summary of trial characteristics

Author/Trial, Year(reference)	Total sample size(n)	Agent(s)	Comparator	Primary outcome	Other outcomes
Motzer RJ et al./NCT00083889,2009 [9]	750	Sunitinib	IFN-α	PFS	ORR,OS,SAE,death
Sternberg CN et al./NCT00334282,2010 [10]	435	Pazopanib	Placebo	PFS	ORR,OS,SAE,death
Gore ME et al.,2010 [11]	1006	IFN-α	IFN-α+IL-2+fluorouracil	OS	ORR,PFS,SAE,death
Escudier B et al.,2010 [12]	649	Bevacizumab+IFN-α	IFN-α+placebo	OS	ORR,PFS,SAE,death
Rini BI et al.,2010 [13]	732	Bevacizumab+IFN-α	IFN-α	OS	ORR,PFS,SAE,death
Rini BI et al.,2014 [14]	791	Temsirolimus+bevacizumab	IFN-α+bevacizumab	PFS	ORR,OS,SAE,death
Escudier B et al./NCT00073307,2009 [15]	903	Sorafinib	Placebo	OS	ORR,PFS,SAE,death
Motzer RJ et al./NCT00720941,2013 [16]	1110	Pazopanib	Sunitinib	PFS	ORR,OS,SAE,death
Motzer RJ et al.,2013 [17]	517	Tivozanib	Sorafinib	PFS	ORR,OS,SAE,death
Hutson TE et al./NCT00920816,2013 [18]	288	Axitinib	Sorafinib	PFS	ORR, SAE,death
Hudes G et al./NCT00065468,2007 [19]	416	IFN-α	Temsirolimus	OS	PFS,SAE,death

Abbreviations: ORR=Objective response rate, PFS=Progression free survival, OS=Over-all survival, SAE=serious adverse events, IL-2=Interleukin-2, IFN=Interferon

### Results from direct comparisons

We did direct comparisons with regard to the efficacy and safety of the therapies for mRCC (Table 2). Among the 11 studies, no repeated comparisons appeared in different studies. Data of progression free survival (PFS) and serious adverse events (SAEs) were reported in every study. Data from 11 selected RCTs showed that efficacy favored tivozanib over sorafinib (SMD = −0.16;95%CI:-0.34,0;*P* < 0.05), bevacizumab+IFN-α over IFN-α+placebo (SMD = −6.85; 95%CI: −7.25,-6.45;*P* < 0.001), bevacizumab+IFN-α over IFN-α (SMD = −0.29; 95%CI: −0.43,-0.14;*P* < 0.05), sunitinib over IFN-α (SMD = −0.61;95%CI: −0.75, −0.46;*P* < 0.05), temsirolimus over IFN-α (SMD = −0.26;95%CI: −0.46,-0.07;*P* < 0.05), pazopanib over placebo (SMD = −7.13;95%CI: −7.64,-6.62;*P* < 0.001), and sorafinib over placebo (SMD = −3.37;95%CI: −3.58,-3.17;*P* < 0.001). With regard to safety, the results showed that safety favored tivozanib over sorafinib (RR = 0.88; 95%CI: 0.78,1.00;*P* < 0.05), IFN-α+placebo over bevacizumab+IFN-α (RR = 1.82;95%CI: 1.35,2.44;*P* < 0.05), IFN-α over bevacizumab+IFN-α (RR = 1.27;95%CI:1.15,1.39;*P* < 0.05), sunitinib over IFN-α (RR = 0.57;95%CI: 0.46,0.70;*P* < 0.05), temsirolimus+bevacizumab over IFN-α+bevacizumab (RR = 1.19;95%CI: 1.01,1.41;*P* < 0.05), temsirolimus over IFN-α (RR = 0.42;95%CI: 0.27,0.67;*P* < 0.05), and placebo over sorafinib (RR = 1.40;95%CI: 1.14,1.73;*P* < 0.05).

**Table 2:** Progression free survival and serious adverse events for efficacy and safety in meta-analyses of direct comparisons between each pair of drugs

	Number of studies	Number of patients	Efficacy		Safety	
			PFS (median,mo.)	SMD(95%CI)	SAE(rate)	RR(95%CI)
Sorafinib *vs*. Axitinib	1	288	6.5*vs*.10.1	0.24 (−0.01,0.48)	24/96*vs*.64/189	0.74 (0.50,1.10)
Tivozanib *vs*. Sorafinib	1	517	11.9*vs*.9.1	**-0.16** **(−0.34,0.00)**	159/260*vs*.179/257	**0.88** **(0.78,1.00)**
Bevacizumab+IFN-α *vs*. IFN-α+Placebo	1	649	10.2*vs*.5.4	**-6.85** **(−7.25,−6.45)**	98/327*vs*.50/304	**1.82** **(1.35,2.44)**
Bevacizumab+ IFN-α *vs*. IFN-	1	732	8.5*vs*.5.2	**-0.29** **(−0.43,−0.14)**	329/450*vs*.231/388	**1.27** **(1.15,1.39)**
IFN-α *vs*. IFN-α+IL-2+fluorouracil	1	1006	5.5*vs*.5.3	−0.02 (−0.15,0.10)	113/502*vs*.131/504	0.87 (0.69,1.08)
Sunitinib *vs*. IFN-α	1	750	11.0*vs*.5.0	**-0.61** **(−0.75, −0.46)**	93/360*vs*.170/375	**0.57** **(0.46,0.70)**
IFN+bevacizumab *vs*. Temsirolimus+bevacizumab	1	791	9.3*vs*.9.1	−0.02 (−0.16,0.12)	177/393*vs*.148/391	**1.19** **(1.01,1.41)**
Temsirolimus *vs*. IFN-α	1	416	5.6*vs*.3.2	**-0.26** **(−0.46,−0.07)**	82/208*vs*.99/200	**0.42** **(0.27,0.67)**
Pazopanib *vs*. Placebo	1	435	9.2*vs*.4.2	**-7.13** **(−7.64,−6.62)**	76/290*vs*.28/145	1.35 (0.93,2.00)
Pazopanib *vs*. Sunitinib	1	1110	8.4*vs*.9.5	0.07 (−0.05,0.19)	230/554*vs*.224/548	0.98 (0.86,1.13)
Sorafinib *vs*. Placebo	1	903	5.5*vs*.2.8	−3.37 (−3.58,−3.17)	154/451*vs*.110/452	1.40 (1.14,1.73)

Abbreviations: SMD=Standardized mean difference, RR=Risk ratio, PFS=Progression free survival, SAE=Serious adverse events, IL-2=Interleukin-2, IFN=Interferon

### Results from network meta-analysis of efficacy and safety

**Figure 3:**
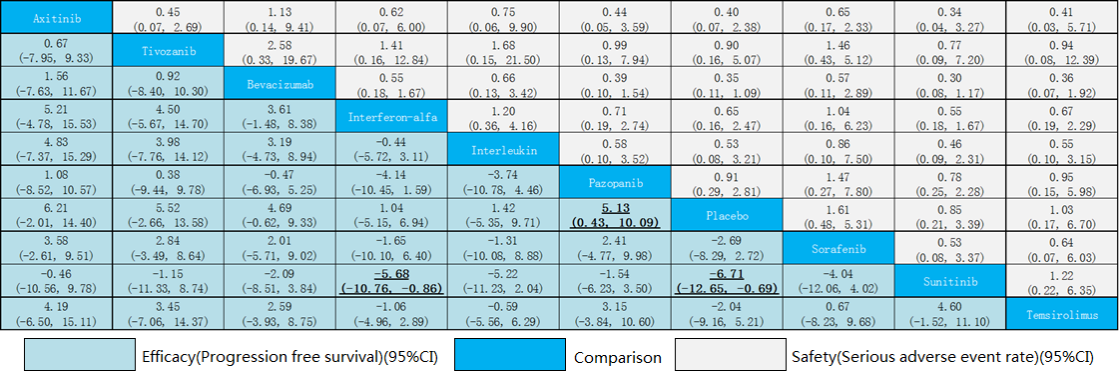
Efficacy and safety of drugs in metastatic renal cell carcinoma

From the eligible studies, 45 indirect comparisons were made. Results of all possible comparisons were expressed with risk ratios, standardized mean difference and 95% credible intervals calculated by Bayesian network meta-analysis. Figure 3 summarizes the results of the network meta-analysis. Only three statistically differences were found in efficacy comparisons. These results demonstrated that sunitinib had a significantly longer PFS than IFN-α (SMD = −5.68; 95%CI: −10.76,-0.86; *P* < 0.001) and placebo (SMD = −6.71; 95%CI: −12.65,-0.79; *P* < 0.001); meanwhile, pazopanib had a significantly longer PFS in comparison to placebo (SMD = 5.13; 95%CI: 0.43, 10.09; *P* < 0.001). Analysis of inconsistency between direct and indirect comparisons indicated that no statistically significant inconsistency was identified in PFS and SAE comparisons (*P* > 0.05).

**Figure 4:**
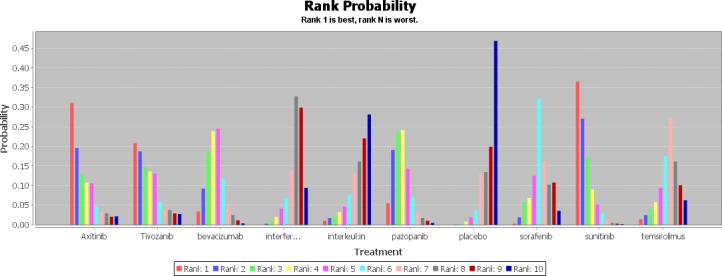
Rank probability of progression free survival

**Figure 5:**
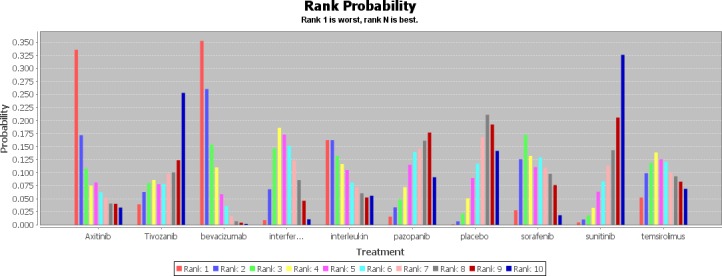
Rank probability of serious adverse events

Values of ranking probability column (Figure 4) indicated that sunitinib had the highest probability of being the best treatment modality in terms of PFS (value = 2.36), followed by axitinib (value = 3.22) and pazopanib (value = 3.69). It was obvious that placebo had the lowest probability of being the best treatment modality (value = 8.83), and IFN-α alone (value = 7.95) and interleukin alone (value = 7.93) also had a rather low probability which were among the last three of the ranking. When it came to SAE (Figure 5), except for placebo (value = 8.06), sunitinib (value = 7.43) and pazopanib (value = 6.80) had the highest probability of being the safest drugs as the first-line treatment. In contrast, bevacizumab combined with IFN-α (value = 2.52) had the lowest probability, followed by axitinib (value = 3.42). This analysis indicated that sunitinib seemed to be the best choice of first-line treatment for patients with mRCC because it had the most favorable balance between efficacy and safety.

## DISCUSSION

In the present study, the results from our indirect comparisons indicated that sunitinib was more efficacious than IFN-α and placebo, which had significant differences between each pair of arms. The overall trend of our network meta-analysis was that targeted therapies had better efficacy and safety which was in accordance with previous studies [20]. Ranking of treatment arms showed that sunitinib had the highest probability of being the best first-line choice for patients with mRCC, because sunitinib not only ranked top in terms of efficacy, but also ranked among the best of safety aspects.

Results from several phase 3 RCTs showed that patients treated with sunitinib as first-line treatment could survive for more than two years [9, 16]. Those patients who had favorable risk factors could survive even much longer [21, 22]. Motzer RJ et al. conducted a randomized phase 3 trial to compare sunitinib and IFN-α in the first-line treatment of patients with mRCC and found that sunitinib demonstrated longer overall survival and progression-free survival [9]. The RECORD-3 trial concluded that everolimus did not yield better results compared with sunitinib as first-line therapy in patients with mRCC and suggested sunitinib be the standard treatment paradigm of first-line therapy [23]. Even though the efficacy of sunitinib has been validated by numerous RCTs, chronic sunitinib treatment still raises questions about its long-term safety. An expanded access trial in mRCC found that there were neither cumulative serious toxicities nor unexpected long-term treatment-related adverse events in patients with wide-ranging disease states to receive sunitinib treatment [24]. Another group of investigators reported a further study of long-term safety for sunitinib using pooled data from 5739 patients with mRCC enrolled in nine prospective clinical trials, including 807 patients treated for ≥2 yr. They found that chronic sunitinib treatment was not associated with new types or increased severity of treatment-related adverse events and toxicity was not cumulative except for hypothyroidism [25].

Targeted therapies are the main treatment modality for mRCC nowadays and targeted drugs are emerging in large numbers, however, it is difficult to establish standard which drug is better than the others in efficacy and safety because head-to-head studies about the medications themselves with each other are limited. The AXIS study was the first RCT to compare two targeted therapies of advanced RCC [26]. The results of this randomized phase 3 trial showed that axitinib resulted in significantly longer PFS compared with sorafenib (6.7*vs*.4.7months, HR = 0.665, 95% CI: 0.544–0.812). When it came to safety, toxic effects in 14 (4%) of 359 patients treated with axitinib resulted in treatment discontinuation and 29 (8%) of 355 patients treated with sorafenib. A recent meta-analysis also concluded that axitinib was superior to sorafenib (HR = 0.46, 95% CI: 0.32-0.68) in prolonging PFS [27]. However, the results from our network meta-analysis indicated that axitinib didn't exhibited a significantly higher efficacy than sorafenib (SMD = 3.58, 95%CI: −2.61, 9.51) as first-line therapy. A safe conclusion can be drawn that axitinib may be a treatment option for second-line therapy of mRCC. Several other head-to-head studies involving two different targeted drugs or one targeted drug *versus* the other cytokine drug as first-line therapies demonstrated significant results. The phase 3 AVOREN and CALGB 90206 trials reported significant PFS benefit of bevacizumab plus IFN-α, which confirmed that the combination of IFN-α and bevacizumab remained as a first-line option of care for patients with mRCC[12, 13]. However, there were significantly more SAEs for bevacizumab plus IFN-α than monotherapy. In our present network meta-analysis, we found that bevacizumab combined with IFN-α had the lowest probability of being the safest therapy for patients with mRCC as first-line option. Consensus has been reached that targeted therapies are more efficacious and safer than cytokine therapies which is in accordance with results from our network meta-analysis [9, 19].

Although sunitinib and other targeted therapies have changed the therapeutic landscape for mRCC, these treatment modalities will achieve limited overall survival after a given agent is no longer effective [28]. Thus, there is an urgent need for treatment options with novel mechanisms of action that could potentially result in improved efficacy and survival advantage. Recent understanding of host-tumor immune interactions has given rise to novel antibodies directed against immune checkpoint proteins which play a vital role in molecular immune response[29]. A randomized, open-label, phase 3 study compared nivolumab (a programmed death 1 checkpoint inhibitor) with everolimus in patients with mRCC who had received previous treatment concluded that overall survival was longer and fewer grade 3 or 4 adverse events occurred with nivolumab [28]. Immune checkpoint inhibitor antibodies are hopeful to be an important therapy for mRCC, even to be first-line option.

There are several limitations should be acknowledged in this study. First of all, our studies included only phase 3 RCTs which resulted in selective bias. Because the quality of the included studies will influence the results of meta-analyses, our analysis was limited to prospective RCTs with sufficient sample size. Second, we did not conduct subgroup analysis according to patient characteristics and prior treatments. As we hoped to measure the overall treatment effect of medications for mRCC, so we included patients meeting the eligibility criteria regardless of their characteristics. Lastly, the failure to acquire several unpublished data led to potential publication bias, even though great efforts had been made to seek for all the available data. Meta-analyses are subject to publication bias because studies with negative results are less likely to be published, therefore resulting in an overestimation.

In conclusion, the results of this network meta-analysis indicated that most of targeted therapies were more efficacious and safer than the other drugs for mRCC. To be specific, sunitinib might be the best choice for clinicians and patients to be considered when both efficacy and safety were balanced. Further information to determine the optimal treatment strategy for mRCC is likely to be from future randomized trials that should examine combined therapies of these active agents. Meanwhile it is important that further comprehensive head-to-head RCTs are carried out for the purpose of assessing the relative efficacy and safety of treatments for mRCC.

## MATERIALS AND METHODS

### Search strategy

We did a systemic search for randomized clinical trials of treatments for mRCC without language restriction until January 31, 2016. All studies were selected according to the search strategy based on Preferred Reporting Items for Systematic Reviews and Meta-analyses criteria (PRISMA) [30]. Two individuals (XC and FZ) searched the following databases and sources independently: PubMed, Embase, the Cochrane Central Register of Controlled Trials (in the Cochrane Library), and unpublished studies were also sought through “clinicaltrials.gov”. The searches combined terms “metastatic renal cell carcinoma”, “advanced renal cell carcinoma”, “randomized controlled trials”, or “RCTs” with the cancer MeSH heading “neoplasms”. We attempted contact with the study authors through email and telephone to obtain full information when necessary.

### Eligibility criteria

We included all the phase 3 RCTs evaluating the therapeutic efficacy and safety of any drug for the first-line treatment of mRCC. We included trials at least with a control intervention, enrolling at least 60 patients with any age, sex or race, and reporting the outcomes of interest (PFS, overall survival (OS), objective response rate (ORR), SAEs, etc.). Non-randomized trials, early results presentations, non-primary studies, animal/laboratory studies, and researches published only in protocols or in abstracts were excluded from present analysis.

### Study selection and data extraction

Two investigators (XC and FZ) independently evaluated the risk of bias and extracted data from eligible trials. If only the standard deviations were missing, they were estimated from p values or with the mean standard deviation of the other included studies [31]. Extracted data were entered into standardized Excel (Microsoft Corp) file and were checked by another author (TL). Any disagreements were resolved by discussion and consensus. To assess the risk of bias of individual trials, we applied the following components recommended by the Cochrane Collaboration: random sequence generation; allocation concealment; blinding of participants, personnel, and outcome assessors (with blinding of at least the outcome assessors required for considering this parameter as low risk of bias); incomplete outcome data; selective outcome reporting; and other sources of bias [32].

### Efficacy and safety outcomes

Efficacy outcomes were primary endpoint including PFS as defined by investigators and secondary endpoints including OS, duration of treatment response, and ORR. Safety outcomes included treatment-related SAEs, which were defined as grade 3 or 4 adverse events, and details on deaths when available.

### Statistical analysis

To incorporate direct comparisons within two trials between two treatments and indirect comparisons from trials having one treatment in common, network meta-analysis methods were applied to all available treatment comparisons to provide the most comprehensive evidence. We compared outcome analyses using risk ratios (RR), standardized mean difference (SMD) and 95% credible intervals (95%CI) with a Bayesian hierarchical random effects model. We used the random effects rather than the fixed effects model because this was likely the most appropriate and conservative analysis to account for differences among trials. We also conducted additional sensitivity analyses by repeating the main computations with the fixed effects method to evaluate the consistency of the results. Potential inconsistency of the network was evaluated by the node split method, measuring agreement between direct and indirect evidence for each split node. Data were analyzed according to the intention to treat principle. Ranking of treatment arms was calculating according to ranking probability column provided by the Bayesian network meta-analysis to determine the best rank. A *p* value < 0.05 was judged as statistically significant, except where otherwise specified. All analyses were conducted with ADDIS version1.16.5 (Copyright ©2013, Gert van Valkenhoef, Joël Kuiper) and R version 2.10.1 (Copyright © 2009, The R Foundation for Statistical Computing, ISBN 3-900051-07-0).
